# *Candidatus* Bartonella mayotimonensis and Endocarditis

**DOI:** 10.3201/eid1603.081673

**Published:** 2010-03

**Authors:** Eleanor Y. Lin, Constantine Tsigrelis, Larry M. Baddour, Hubert Lepidi, Jean-Marc Rolain, Robin Patel, Didier Raoult

**Affiliations:** Mayo Clinic, Rochester, Minnesota, USA (E. Y. Lin, C. Tsigrelis, L.M. Baddour, R. Patel); Université de la Méditerranée, Marseille, France (H. Lepidi, J.M. Rolain, D. Raoult); 1 Current affiliation: Massachusetts General Hospital, Boston, Massachusetts, USA

**Keywords:** Endocarditis, Bartonella, Candidatus Bartonella mayotimonensis, aortic valve, bacteria, dispatch

## Abstract

We describe a new *Bartonella* species for which we propose the name *Candidatus* Bartonella mayotimonensis. It was isolated from native aortic valve tissue of a person with infective endocarditis. The new species was identified by using PCR amplification and sequencing of 5 genes (16S rRNA gene, *ftsZ*, *rpoB*, *gltA*, and internal transcribed spacer region).

*Bartonella* species are small, fastidious, gram-negative, intracellular bacteria that cause culture-negative infective endocarditis. Six species have been documented to cause endocarditis in humans: *B*. *quintana* (*1*), *B*. *henselae* (*2*), *B*. *elizabethae* (*3*), *B*. *vinsonii* subsp. *berkhoffii* (*4*), *B*. *koehlerae* (*5*), and *B*. *alsatica* (*6*). We report a case of culture-negative endocarditis caused by a new *Bartonella* species, for which we propose the name *Candidatus* Bartonella mayotimonensis.

## The Patient

A 59-year-old man was initially hospitalized at Sartori Memorial Hospital (Cedar Falls, IA, USA) from April 14 through 19, 2008, for progressive shortness of breath, weight loss, fatigue, and altered mental status. He was then transferred to the Mayo Clinic (Rochester, MN, USA). Physical examination identified a new diastolic heart murmur. He was afebrile and did not have peripheral stigmata of endocarditis. Two sets of blood cultures obtained before antimicrobial drug therapy showed negative results for all bacteria tested after 5 days of incubation. A transesophageal echocardiogram showed a bicuspid aortic valve, mobile components on the left cusp of the aortic valve suggesting vegetations, and a 5.3-cm ascending aortic aneurysm. Empiric antimicrobial drug therapy, including vancomycin and ceftriaxone, was initiated. Subsequently, acute renal dysfunction, possibly secondary to vancomycin exposure, developed in the patient.

The patient lived alone on a farm in Iowa, USA, and had not had recent exposure to animals. However, he had observed murine fecal droppings in his house and mice on the farm. He had had a house cat for 18 years until its death a few years before his hospitalization and had intermittent contact with cats when he visited his daughter.

Serum immunoglobulin G titers were positive for *B*. *henselae* and *B*. *quintana* (>1,024). Oral doxycycline and rifampin were prescribed for treatment of presumed *Bartonella* endocarditis. Gentamicin was not administered because of development of the acute renal dysfunction. Two weeks later, he underwent aortic valve and aortic root replacement. Results of gram staining, acid-fast staining, fungal staining, anaerobic bacterial culture, aerobic bacterial culture, mycobacterial culture, and fungal culture on resected aortic valve tissue were negative for *Bartonella* species.

PCR performed at the Mayo Clinic on resected aortic valve tissue detected part of the citrate synthase gene (*gltA*) of *Bartonella* species. However, the melting temperature was not characteristic of *B*. *quintana* or *B*. *henselae* (*7*). Oral doxycycline and rifampin were continued for 12 weeks after aortic valve resection. The patient was well and had no signs of relapsing infection at a follow-up visit 11 months after valve surgery.

Aortic valve tissue and serum were tested at the Unité des Rickettsies, Marseille, France. *B*. *quintana* Oklahoma, *B*. *henselae* Houston (ATCC 49882), *B*. *vinsonii* subsp. *berkhoffii* (URBVAIE25), *B*. *vinsonii* subsp. *arupensis* (ATCC 700727), and *B*. *alsatica* (CIP 105477 T) strains were used for immunofluorescent assays and Western blotting (*6*). Valve tissue was injected into human endothelial cells in a shell vial assay and onto Columbia 5% sheep blood agar plates and incubated at 37°C in an atmosphere of 5% CO_2_ as described (*6*).

A *Bartonella* species was detected in a shell vial by immunofluorescence after 15 days of culture; identification was confirmed by PCR. DNA was extracted from valve specimen and injected cells by using the QIAamp Tissue Kit (QIAGEN, Hilden, Germany). DNA was used as a template in a genus *Bartonella* Lightcycler assay with primers and a Taqman probe specific for the internal transcribed spacer (ITS) gene (*6*) and in standard PCR assays specific for the 16S rRNA, ITS, *rpoB*, *gltA*, and *ftsZ* genes (*8*). Sequences from both DNA strands were determined twice for all PCR products. These products were resolved in an ABI 3100 automated sequencer (PerkinElmer, Waltham, MA, USA). Sterile water was used as a negative control in each assay. Percentages of similarity among sequences were determined by using MEGA 2.1 software (*9*). Phylogenetic relationships among *Bartonella* strains were inferred from concatenated sequences by using MEGA 2.1 software (*9*).

Surgically resected aortic valve tissues were fixed in formalin, embedded in paraffin, and sectioned to a thickness of 5 μm. Sections were stained with periodic acid–Schiff, Giemsa, Gram, Grocott-Gomori methenamine silver, and Warthin-Starry stains. Immunohistochemical analysis was performed by using a procedure described elsewhere (*10*) and polyclonal antibody against *B*. *vinsonii* at a dilution of 1:1,000.

**Figure 1:**
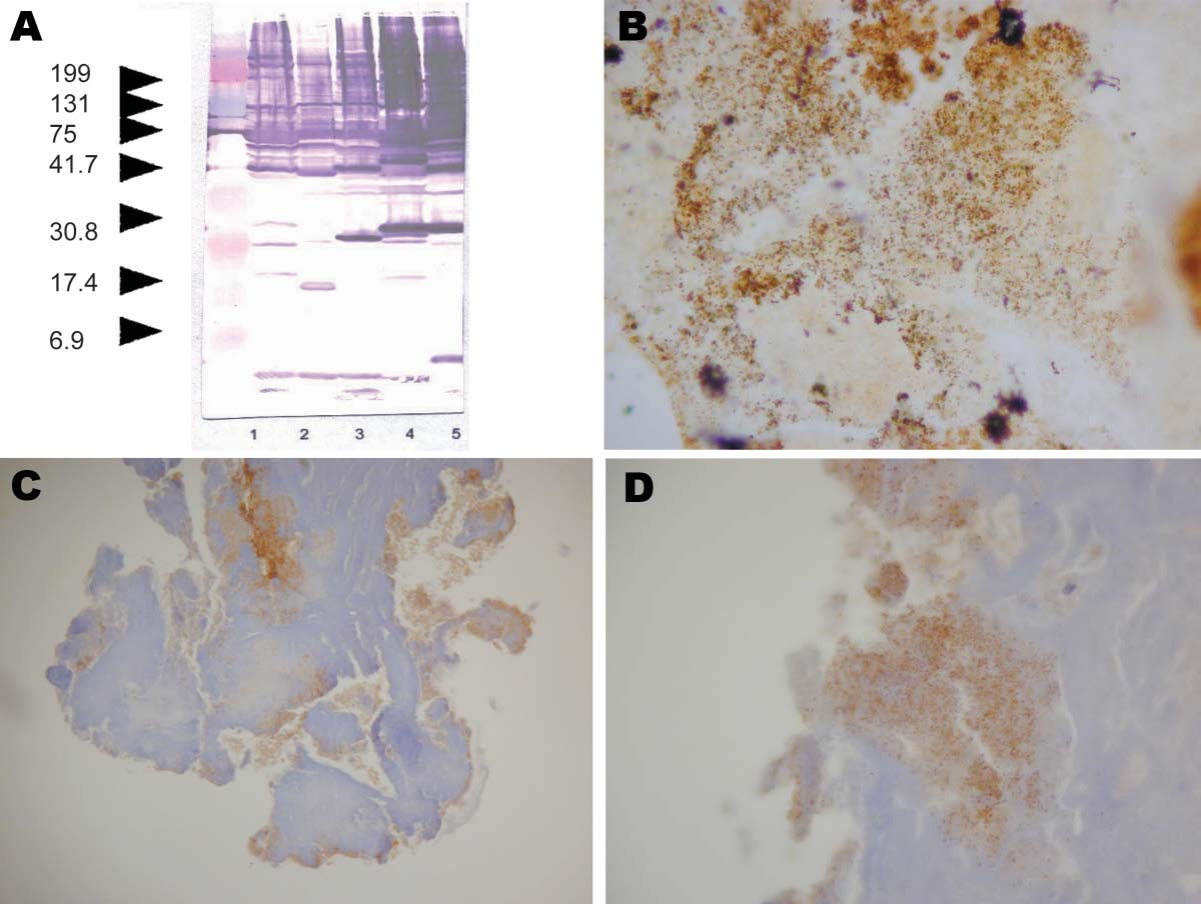
A) Western blot of serum sample from patient infected with *Candidatus* Bartonella mayotimonensis. Left lane, Molecular mass standard; lane 1, *Bartonella* *quintana*; lane 2, *B*. *henselae*; lane 3, *B*. *elizabethae*; lane 4, *B*. *vinsonii* subsp. *berkhoffii*; lane 5, *B*. *alsatica*. Values on the left are in kilobases. B) Numerous darkly stained bacilli consistent with *Bartonella* species organized in clusters in the valvular vegetation (Warthin-Starry stain; original magnification ×400). C and D) Bacteria detected by immunohistochemical analysis of an extracellular location inside the valvular vegetation (polyclonal antibody against *B. vinsonii* subsp. *berkhoffii*, Warthin-Starry stain and hematoxylin counterstain; original magnification ×100 in C and ×400 in D).

**Figure 2:**
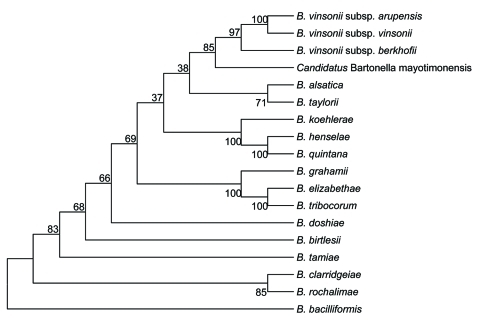
Phylogenetic tree showing the position of *Candidatus* Bartonella mayotimonensis among members of the genus *Bartonella* based on comparisons of concatenated sequences of the 16S rRNA gene, the citrate synthase gene *gltA*, the RNA polymerase β-subunit gene *rpoB*, the cell division gene *ftsZ*, and the 16S–23S rRNA internal transcribed spacer region sequences. The tree was constructed by using the neighbor-joining method and a maximum-likelihood–based distance algorithm. Numbers on branches indicate bootstrap values derived from 500 replications.

Serum samples showed immunoglobulin G endpoint titers of 50 against all *Bartonella* species tested by immunofluorescent assay. Western blot results were positive and characteristic of *Bartonella* infection (Figure 1, panel A). Results of PCR (*Bartonella* genus Lightcycler assay and standard PCR for cardiac valve) and cell culture were positive, and amplification products of the expected size were obtained. Among known validated species, sequences obtained shared 99.1% (1,438/1,445 bp) homology with *B*. *tribocorum*, *B*. *henselae*, and *B*. *vinsonii* for the 16S rRNA gene, 89.5% homology with *B*. *grahamii* for the ITS gene, 93.4% homology with *B*. *vinsonii* subsp. *berkhoffii* for *rpoB*, 91.7% homology with *B*. *vinsonii* subsp. *berkhoffii* for *ftsZ*, and 92.5% homology with *B*. *vinsonii* strain Baker for *gltA*. The phylogenetic position of *Candidatus* B. mayotimonensis among members of the genus *Bartonella* based on comparisons of concatenated sequences of the 5 genes is shown in Figure 2. Sequences of *gltA,* 16S rDNA, *ftsZ,* ITS, and *rpoB* were deposited in GenBank under accession nos. FJ376732–FJ376736.

Histologic analysis of resected aortic valve showed infective endocarditis with vegetation containing microorganisms that stained with Warthin-Starry and Giemsa. Warthin-Starry staining showed darkly stained bacilli consistent with *Bartonella* species (Figure 1, panel B). Results of staining with periodic acid–Schiff, Gram, and Grocott-Gomori methenamine silver were negative. Immunohistochemical analysis detected bacteria in valvular vegetations in a location superimposable with that detected by Warthin-Starry staining (Figure 1, panels C, D).

## Conclusions

We isolated a new *Bartonella* species and propose that it be named *Candidatus* Bartonella mayotimonensis to recognize the contributing institutions (Mayo Clinic and Hôpital de la Timone, Marseille, France). This is the seventh *Bartonella* species documented to cause infective endocarditis in humans.

The reservoir of *Candidatus* B. mayotimonensis has yet to be determined. Different *Bartonella* species have been isolated from a variety of mammals, and each species is highly adapted to its reservoir host (*11**,**12*). The domestic cat is the primary mammalian reservoir for *B*. *henselae* (*13*). Other *Bartonella* species have been found in mammalian hosts, including rats (*B*. *elizabethae*), dogs and coyotes (*B*. *vinsonii* subsp. *berkhoffii*), cats (*B*. *koehlerae*), humans (*B*. *bacilliformis* and *B*. *quintana*), moles (*B*. *talpae*), voles (*B*. *vinsonii* subsp. *vinsonii*), cows (*B*. *bovis* [*weissii*]), deer (*B*. *schoenbuchensis*), and rabbits (*B*. *alsatica*) (*3**–**6**,**12**,**14**,**15*). Our patient had direct exposure to mice on his farm and also had intermittent contact with cats while visiting his daughter. Additional investigations are needed to determine the reservoir(s) and vector(s) for this novel bacterium.

The immunofluorescent assay, the current serologic method for diagnosis of *Bartonella* infection, does not distinguish among *Bartonella* species. Only Western blot analysis and cross-adsorption enable serologic identification of species. PCR and culture are critical when a *Bartonella* species is identified for the first time as a human pathogen. Newly encountered *Bartonella* strains should be considered a new species if a 327-bp *glt*A fragment shares <96.0% sequence similarity with those of validated species, and if an 825-bp *rpo*B fragment shares <95.4% sequence similarity with those of validated species as reported in the current case (*8*).

This case reinforces the hypothesis that any *Bartonella* species can cause human infection, including culture-negative endocarditis. *Candidatus* B. mayotimonensis should be added to the list of human pathogens that can cause culture-negative endocarditis.
